# Automated Cell Selection Using Support Vector Machine for Application to Spectral Nanocytology

**DOI:** 10.1155/2016/6090912

**Published:** 2016-01-19

**Authors:** Qin Miao, Justin Derbas, Aya Eid, Hariharan Subramanian, Vadim Backman

**Affiliations:** ^1^Biomedical Engineering Department, Northwestern University, Evanston, IL 60208, USA; ^2^NanoCytomics LLC, 1801 Maple Avenue, Evanston, IL 60201, USA

## Abstract

Partial wave spectroscopy (PWS) enables quantification of the statistical properties of cell structures at the nanoscale, which has been used to identify patients harboring premalignant tumors by interrogating easily accessible sites distant from location of the lesion. Due to its high sensitivity, cells that are well preserved need to be selected from the smear images for further analysis. To date, such cell selection has been done manually. This is time-consuming, is labor-intensive, is vulnerable to bias, and has considerable inter- and intraoperator variability. In this study, we developed a classification scheme to identify and remove the corrupted cells or debris that are of no diagnostic value from raw smear images. The slide of smear sample is digitized by acquiring and stitching low-magnification transmission. Objects are then extracted from these images through segmentation algorithms. A training-set is created by manually classifying objects as suitable or unsuitable. A feature-set is created by quantifying a large number of features for each object. The training-set and feature-set are used to train a selection algorithm using Support Vector Machine (SVM) classifiers. We show that the selection algorithm achieves an error rate of 93% with a sensitivity of 95%.

## 1. Introduction

Lung cancer remains the leading cause of cancer mortality in the United States, resulting in more deaths than breast, prostate, and colorectal cancers combined. In 2015, about 158,000 Americans are expected to die from lung cancer, accounting for 27% of all cancer deaths. Even though it can be effectively managed by surgery at an early stage, most patients do not show noticeable symptoms until the disease is at an incurable stage. As a result, over half of people with lung cancer die within one year of being diagnosed, and the five-year survival rate (17.8%) is lower than many other leading cancers [1]. Smokers comprises about 90% of lung cancer patients [2], which makes early screening an effective tool for prevention. However, past attempts to screen for lung cancer, such as sputum cytology and chest X-ray, have failed to provide clinically satisfactory results for population screening due to suboptimal efficacy, equivocal survival benefit, and numerous false positives affecting cost-effectiveness [3]. Therefore, an accurate, minimally intrusive prescreening method is urgently needed. It has been shown that the environmental and genetic pathogenic factors that cause neoplastic lesions in the lung will affect the entire respiratory and digestive tract mucosa, which is known as field carcinogenesis [4]. In particular, the buccal epithelium represents an attractive target for field effect detection of lung carcinogenesis [5].

Recently, a novel optical approach called nanocytology based on partial wave spectroscopic (PWS) microscopy has been developed, which can quantify statistical properties of cellular structures at the nanoscale [6–8]. PWS is much more sensitive than traditional wide-field microscopic examination and is capable of detecting malignancies even before any visible morphological changes [9]. PWS microscopy has been shown to improve the accuracy of early screening for a number of different types of cancer [10–13]. In the PWS system, low-spatially coherent light illuminates the sample and the backscattered photons are collected. The spectral fluctuations in the backscattering spectra are analyzed for measurement of nanostructures inside the sample. This enables the quantification of the statistical properties of the spatial refractive index variations at any length scale, including those well below the diffraction limit. The statistical parameter called “disorder strength” can be determined from this analysis and used as a diagnostic biomarker for cancer. An image of the distribution of disorder strength is generated and from these two-dimensional (2D) images several statistical parameters, such as mean disorder strength, can be extracted for diagnosis.

**Figure 1:**
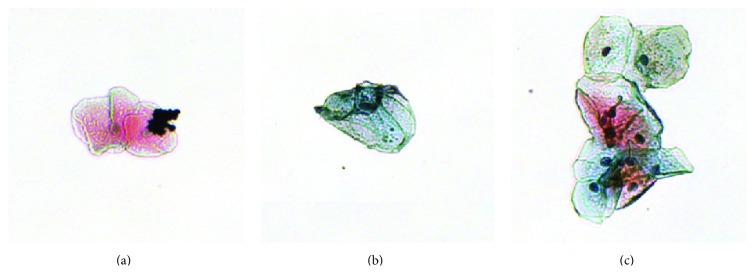
Examples of unwanted objects encountered in prepared samples. (a) Debris-covered cells; (b) folded cells; (c) overlapping cells.

Since the PWS signal is very sensitive, in order to obtain accurate and precise results it is critical to select a subset of suitable cells from the raw images. The suitable cells are defined as cells that are isolated, not folded, and not covered with debris. Some common artifacts are shown in Figure 1.

In conventional cytology, many automated smear analysis methods have been developed. Such automated analysis consists of segmentation of structures inside the cell and classification using features computed from the segmented region of interest. The early work in segmentation used grayscale thresholding [14], but recently more complex methods have been developed [15–17]. Features can be derived from these segmented regions of interest and used to train classifiers for diagnosis. Many approaches to the automated analysis of features have been developed [18–23].

Although techniques for automated smear analysis exist, there are several factors which led to the current study being undertaken. First, the cell selection criteria for PWS microscopy is unique and different from conventional cytology. In conventional cytology, a single cell is needed for analysis, but the cell needs not be isolated from neighboring cells. This is because the diagnosis in the cytology depends on the morphology of the cells, for example, nucleus/cytoplasm ratio [24]. As a result, even if the cell is embedded in a cell cluster, as long as the shape of a single cell can be identified, it can be used for diagnosis. However, this is not the case for PWS microscopy. In PWS, isolated cells are required so that accurate and useful statistical information can be identified and analyzed. Second, in real practice, many cells and nucleus are corrupted by different artifacts, such as debris and distorted cells, but the previously developed segmentation algorithms for cytology fail to address these practical problems since they are intrinsically created to avoid picking up unwanted objects. As a result, there will in most cases be much debris among the segmented “nucleus” and “cells.” If such unwanted cells are subject to analysis, it will provide unreliable results and cause great difficulties in designing a system with low false positive rates. Third, to date cell selection for PWS system has been a manual process. The selections are subjective and depend on the experience of each operator. As a result, the selection results are subject to bias and imprecise due to these biases. The remedy is to automate the cell selection process by using quantitative morphological features.

There are a large variety of different classification methods that have been developed and found applications in different scientific fields [25–29]. Among these algorithms, Support Vector Machines (SVM) are well-known for their high generalization ability in solving linear and nonlinear classification problems and have shown a high classification performance on many applications [30–35]. Therefore, an SVM is used as the classification algorithm in the present study.

In this study, an automated cell selection method has been developed for the PWS system to select suitable cells from buccal smear samples. A set of features are extracted from cells, and the training dataset for classifiers is formed by extracting these features from 1000 cells that are manually labeled by an experienced operator. The trained classifier is found to be able to effectively remove unsuitable objects from raw buccal smears.

## 2. Materials and Methods

### 2.1. Imaging System

The PWS system is built on a commercial Nikon microscope (Eclipse Ni-E). The data used in this study is taken under the transmission illumination mode, which is independent of the PWS signal acquisition. Since Kohler illumination is used in the system the illumination on the sample plane is uniform. Transmission images are taken by a CCD (ORCA-Flash2.8, Hamamatsu) using a 10x objective lens (CFI Plan Fluor DLL 10x, NA 0.3). The whole slide is imaged by automatically scanning the sample stage. These images are then tiled together to form a complete image of the slide, herein called a slide-map. The cells on the slide-map will be analyzed using the classifiers developed in this study and only those classified as suitable cells will be further analyzed for diagnosis.

### 2.2. Classification Technique

The automated algorithm for selection of suitable cells was benchmarked against manual cell selection by experienced operators. The definition of a suitable cell is subjective and formed by experimental experience. Automated cell selection involves numerically representing morphology markers that distinguish suitable and unsuitable cells for further cytological analysis. These markers were used as input features to a classifier that provides a statistical score determining the probability of suitability. Features were defined to represent the morphological characteristics known to experienced operators which are used to distinguish suitable cells. The quality of a cell may be described by many characteristics, including isolation, size, shape, color, roughness, and folding. A total of 100 features were computed for raw cell images in the study. The set of features used in the study is summarized in Table 1.

### 2.3. Sample Preparations

The sample preparation follows the liquid-based cytology method [38–40]. Buccal cells were brushed from the patient's cheek and rinsed in the vial of collection fluid. After the samples are transferred to the lab, the samples are deposited onto a glass slide. The cells on the slide are then fixed in 95% ethanol, rinsed in water, and then stained using the commonly used Papanicolaou staining protocol (contains hematoxylin and CytoStain). Finally, the slides are dipped in ethanol of different concentrations and dried for imaging.

### 2.4. Slide-Map Images

**Figure 2:**
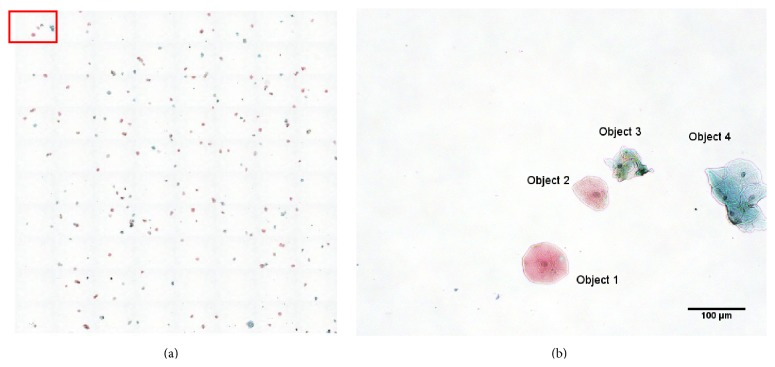
(a) An example of a slide-map, the red rectangle enclosed region is shown in (b); (b) a tile from the slide-map. There are four objects detected in (b), in which objects 1 and 2 are considered suitable cells and objects 3 and 4 are overlapped cells and considered unsuitable (objects).

The whole slide-mapping images are imaged using a low-magnification (10x) objective lens under transmission illumination. This is accomplished using an algorithm that rapidly collects many low-resolution images and tiles them together to create a full image of the slide. A user defines the bounds of the region to be mapped by specifying the positions of two diagonal corners. The algorithm then calculates the number of images required to map the entire region specified based on a pixels-to-micron conversion factor specific to the objective and imaging sensor used. The region is then raster scanned, and an image is acquired at each *x* and *y* position in order to make a complete image of the region without gaps or overlaps. Then all the images are tiled together to form a complete image of the entire region.  Figure 2 shows an example of a slide-map.

### 2.5. Training and Testing Database

Manual cell selection was performed by experienced operators using homemade software. A slide-map may contain fluctuations in mean luminance, which can interfere with subsequent segmentation. Hence the slide-map is first corrected for uneven illumination by using a blank background image. The objects in the slide-map are segmented from the background. Since stain is taken up by the cell, typically there is a sharp gradient that separates the background grey level from that of the rim of the cells. Each tile was 1024 × 1280 pixels in size and was reduced to half resolution scale using bicubic interpolation to reduce computation time without dramatically compromising the image quality. *H*-minima and *H*-maxima transforms were used with a fixed threshold to flatten low contrast pixels. Sobel and log edge detectors were used to generate an edge image. Finally, morphological operations were used to fill in the image and clean it up, resulting in a clean binary mask localizing mid- to high-contrast objects. Each object inside the slide-map is presented to the operator one at a time. The operator then labels the detected objects as either suitable or unsuitable. The selection results are stored in a file that has the boundary coordinates and suitability of the objects. These manually selected objects were used as the training and testing data for the classifier. The training database consists of 1000 objects and the testing database has 360 objects, half of which are suitable cells. All the features except those for color are calculated from grayscale images, which are obtained by taking the average of the three channels in the raw slide-map.

### 2.6. Classifier Development

**Figure 3:**
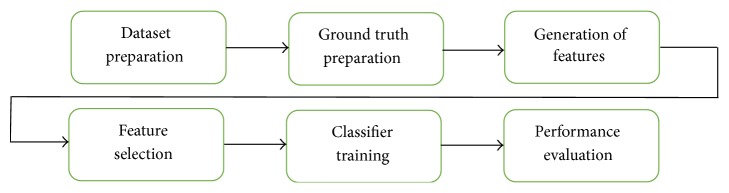
Steps for cell selection classifier development.

Figure 3 shows the steps taken to develop the classifier for the automated cell selection. Dataset preparation along with the ground truth preparation has been explained in the last section. After generating all the proposed features, the next step is to select the best subset of features for the classification. In this study, feature selection is independent of classifier.

### 2.7. Feature Selection

The feature selection step consists of a search strategy and evaluation function. In the search strategy step, subsets of features from the original feature-set are generated. An evaluation function is used to compare the performance of these selected feature subsets. Interclass distance measures are usually chosen as an evaluation function. The most widely used interclass measures, such as Bhattacharyya distance, all assume that the data follows a known distribution. However, most of the features in the study have complex or unknown statistical properties. Nonparametric feature selection assures no prior assumptions were made regarding the statistical distribution that characterize the features. For this reason, a nonparametric separability measure is used here to evaluate the generated feature subsets. A modified Fisher's criterion is employed in the study [41]. The improvement over Fisher's criteria is due to putting weights on every sample to compute the weighted means and defining new nonparametric between-class and within-class scatter matrices. Since in this study there are only two classes, the nonparametric between-class scatter matrix is defined as

(1) Sb=∑i=12Pi∑j=1j≠i2∑k=1niλki,jnixki−Mjxkixki−MjxkiT,

where *x*_*k*_^*i*^ is the *k*th sample from class *I*, *P*_*i*_ is the prior probability of class *i*, and *n*_*i*_ is the sample size of class *i*. The scatter matrix weight *λ*_*k*_^(*i*, *j*)^ is defined as

(2) $$\begin{array}{c} \lambda_{k}^{\left(i,j\right)}=\frac{d{\left(x_{k}^{i},M_{j}\left(x_{k}^{i}\right)\right)}^{-1}}{\sum_{l=1}^{n_{j}}d{\left(x_{l}^{i},M_{j}\left(x_{l}^{i}\right)\right)}^{-1}}, \end{array}$$

where *d*(*a*, *b*) means the Euclidean distance between *a* and *b*. *M*_*j*_(*x*_*k*_^*i*^) is the weighted mean of *x*_*k*_^*i*^ in class *j* and defined as

(3) $$\begin{array}{c} M_{j}\left(\;x_{k}^{i}\;\right)=\overset{n_{i}}{\underset{l=1}{\sum }}w_{kl}^{\left(i,j\right)}x_{l}^{i}, \end{array}$$

where

(4) $$\begin{array}{c} w_{kl}^{\left(i,j\right)}=\frac{d{\left(x_{k}^{i},x_{l}^{j}\right)}^{-1}}{\sum_{l=1}^{n_{i}}d{\left(x_{k}^{i},x_{l}^{j}\right)}^{-1}}. \end{array}$$

The nonparametric within-class scatter matrix is defined as

(5) $$\begin{array}{c} S_{w}=\overset{2}{\underset{i=1}{\sum }}P_{i}\overset{n_{i}}{\underset{k=1}{\sum }}\frac{\lambda_{k}^{\left(i,i\right)}}{n_{i}}\left(\;x_{k}^{i}-M_{i}\left(\;x_{k}^{i}\;\right)\;\right){\left(\;x_{k}^{i}-M_{i}\left(\;x_{k}^{i}\;\right)\;\right)}^{T}. \end{array}$$

The performance of the feature subsets is then compared by using the evaluation criteria:

(6) $$\begin{array}{c} F=\left|\;S_{b}\;\right|S_{w}^{-1}. \end{array}$$

Search of the feature-set was done in an independent way for the classifier using an increasing number of features in a stepwise fashion [42]. For each step, the best feature that satisfies the evaluation function is included into the current feature-set. The algorithm also verifies the possibility of improvement of the evaluation function if one feature is excluded. The step is repeated until the desired number of features is reached.

### 2.8. Classifier Training

After the selection of optimal feature-sets, the data is used for classifier training. Since the data is not linearly separable and features are heterogeneous, Support Vector Machine (SVM) is used for the classification. It has been shown in several studies that classification by an SVM is better in performance and tolerant to irrelevant and interdependent features than other nonparametric classifiers [43–45]. Gaussian radial basis function is used in this study. Penalty factor and scale factor are optimized by 10-fold cross-validation [46].

### 2.9. Performance Evaluation

We selected classification techniques to reduce overspecialization to training data [46]. Overspecialization occurs when the classifier fits around training datasets in ways that do not reflect the true characteristics of the feature distributions. In this study, the training results are checked for overtraining using the leave-out-one method of cross-validation [47]. A receiver operating characteristic ROC curve and sensitivities are calculated to evaluate the performance of the classifiers. Confidence intervals are calculated using the bootstrap method [48].

## 3. Results

**Figure 4:**
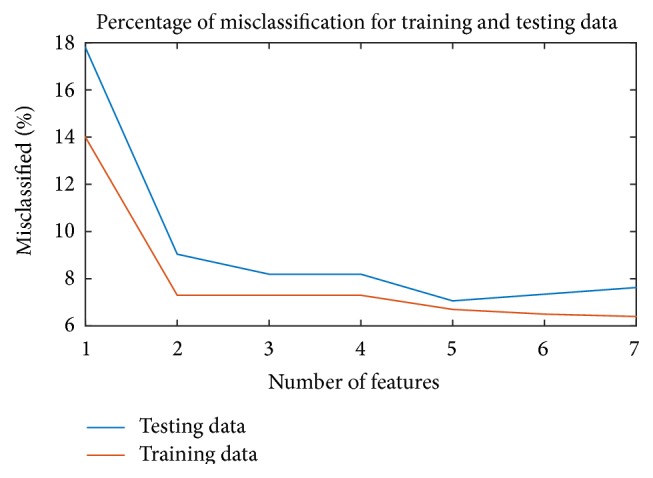
Error rate of classifier on training and testing dataset.

Figure 4 shows the performance (error rate) of the trained classifier versus number of features tested on the whole training (red) and testing (blue) datasets. The classification error is defined as the number of incorrectly classified cells divided by the total number of cells. The trend of Figure 4 shows that an asymptotic error rate is achieved for the current classifier using seven features. Cross-validation is used to verify that the classifiers are not overtrained. Observations are made of the differences between the error rate using training data and that using testing data versus the number of features used for classification. Overtraining is indicated if this difference systematically increases with an increasing number of features. It can be seen that no systematic increase appears for the classifiers, indicating that the classifiers are not overtrained.

Table 2 shows the top ranked features used for the classifiers. These features represent different aspects of the cells: shape (eccentricity, solidity); histogram features (variance, kurtosis); textures (entropy, energy, and variance of Gabor-filtered image). All these features are calculated from grayscale images that are obtained by taking the average of three color channels in the raw data. These features also agree with the empirical definition of suitable and unsuitable objects. The suitable cells usually are round and have a smooth texture, while the folded, overlapping cells or debris tends to have more irregular shapes and more variation in intensity distribution across the cells.

Table 3 shows a comparison of calculated features between a suitable and an unsuitable cell. The unsuitable cells usually have an odd shape, so they typically have high eccentricity and low solidity. Similarly, the suitable cells are usually smooth while the unsuitable cells have coarser texture, which are indicated by other textural features.

**Figure 5:**
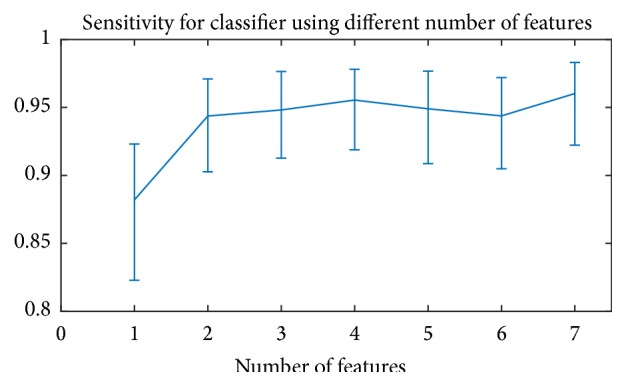
Sensitivity versus number of features. Error bar represents 5%–95% confidence intervals.

Figure 5 shows the sensitivity of the classifiers versus number of features. We can see that the asymptotic sensitivity reaches about 95%.

**Figure 6:**
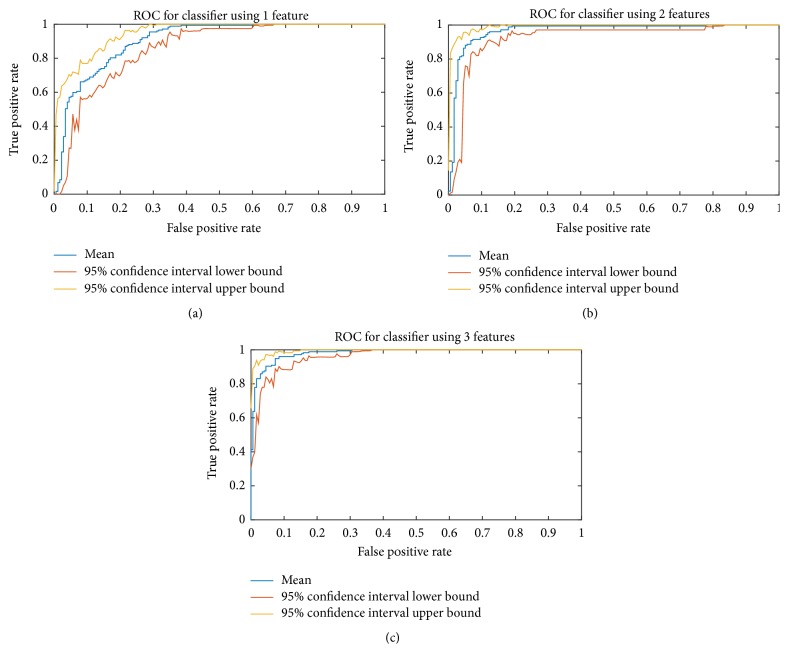
ROC curve for classifiers using 1 (a), 2 (b), and 3 (c) features.

A receiver operating characteristic (ROC) curve is a metric that illustrates the performance of classifiers [49, 50]. In an ROC curve, the true positive rate is plotted versus the false positive rate. ROC curve is calculated for the classifier to evaluate its effectiveness in distinguishing the two classes.  Figure 6 shows the ROC curves for the trained classifier using 1–3 features. In the Figures 6(a)–6(c), blue curves represent the mean value of the ROC curve, while red and yellow curves represent the 5% and 95% confidence interval. As the number of introduced features increases, the curves bow more to the left of the diagonal line, which indicates the increased accuracy of the classifier.

**Figure 7:**
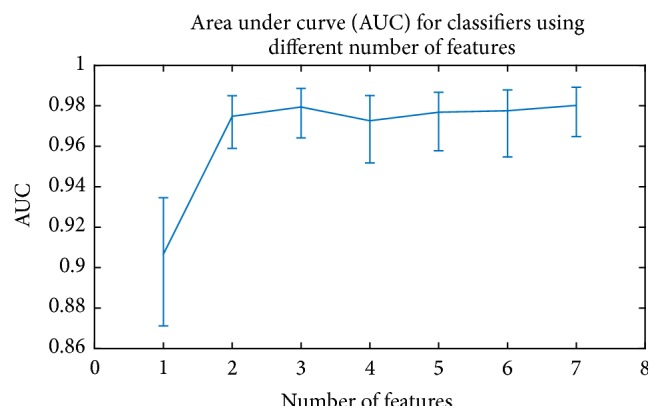
AUC value for classifiers using different numbers of features.

The area under the ROC curve (AUC) (the *c*-statistic) can be used to quantitatively measure the performance of the classifiers [51]. It represents the probability that the classifier will rank a randomly chosen positive instance higher than a randomly chosen negative instance.  Figure 7 shows AUC values for the classifiers using different numbers of features. The asymptotic value for AUC is about 0.98, which indicates that the classifier is effective in distinguishing the two classes of objects.

## 4. Discussion

In conventional cytological practice, screening, and diagnosis are based on observing the morphological changes of cells when they are transformed into malignant cells. For example, the cell nucleus becomes larger and the cytoplasm becomes relatively smaller so that the nuclear cytoplasm ratio changes. The texture of the chromatin is also an important factor, since the chromatin distribution in the nucleus becomes coarser and irregularly distributed. However, it is often already too late for patients when these morphological changes start to appear. As stated previously, over half of people with lung cancer die within one year of being diagnosed, because the samples from most patients do not show noticeable abnormalities until it is already at an incurable stage. If abnormalities can be detected before the noticeable morphological changes, then many patients can be saved by surgical intervention at an early stage. PWS microscopy detects the nanoscale statistical properties inside cells and has been shown to distinguish abnormal cells even before the morphological features used in conventional cytology begin to appear. PWS analysis utilizes the concept of field carcinogenesis, which states that the genetic and epigenetic alterations in early cancer stage occur not only at the neoplastic focus but also more diffusely throughout the affected organ. For example, PWS has been shown to be able to screen for lung cancer by assessing the check cells based on genetic and epigenetic data that suggests that buccal epithelial cells are altered in lung field carcinogenesis. These buccal cell samples already possess alterations at the molecular level but appear normal according to conventional cytological standards. Therefore, the cell selection criteria used in conventional cytology cannot be applied in PWS analysis, and the previously published methods that are based on these criteria cannot be used in PWS analysis either. One major difference is that PWS requires single isolated cells while in conventional cytology only a single cell is needed, even if it is inside a clump of cells. Clinical studies have been conducted to compare the performance of PWS microscopy using cells with optimal characteristics (isolated) and suboptimal characteristics (overlapping). Effect size is calculated to quantify the performance, which is defined as difference in means over the square root of the sum of the variances. It quantifies the difference between the controls and cancers while taking into account the standard deviations. It turns out that the effect size for analysis using suitable cells (isolated, smooth) is 108.5% while it is only 62% for analysis using unsuitable cells. As a result, while the previously published cell selection methods put much effort into segmenting single cell from cell clumps, we focus on finding isolated cells that are not folded, are not overlapped by neighboring cells, are not covered with debris, and have smooth appearance. These qualitative descriptions of criteria are quantitatively identified in this study. Since the samples in PWS analysis are either normal cells or cells at early stage that have not shown the noticeable morphological changes, all the samples uses the same cell selection procedure.

In SVM methods, kernel functions are used to map the input data vector into higher dimensional spaces. In the new space, the mapped feature vectors can be linearly separable or have improved separability. Some commonly used kernel functions are shown in Table 4, in which 〈*x*_*i*_, *x*_*j*_〉 represents the inner product for two feature vectors and ‖*x*_*i*_ − *x*_*j*_‖ is the Euclidean distance between them.

**Figure 8:**
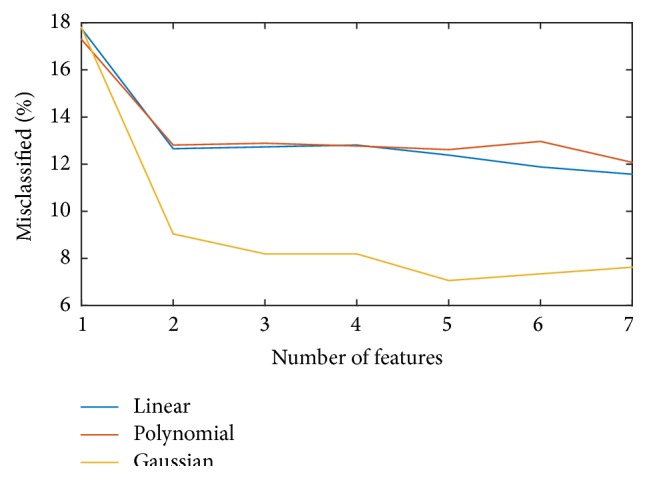
Error rates of classifiers with linear, polynomial, and Gaussian kernels.

The performance of an SVM classifier is dependent on the choice of kernel function. In order to find the best kernel for the current application, three classifiers using different kernel functions are trained by the same training data and compared by testing it on the same testing dataset. During training, the best set of parameters for each kernel function is searched by applying a 4-fold cross-validation method.  Figure 8 shows the classification accuracy results for all three kernels. It can be seen that the SVM classifier using radial basis function provides the best results; thus a Gaussian radial basis function is chosen for this study.

**Figure 9:**
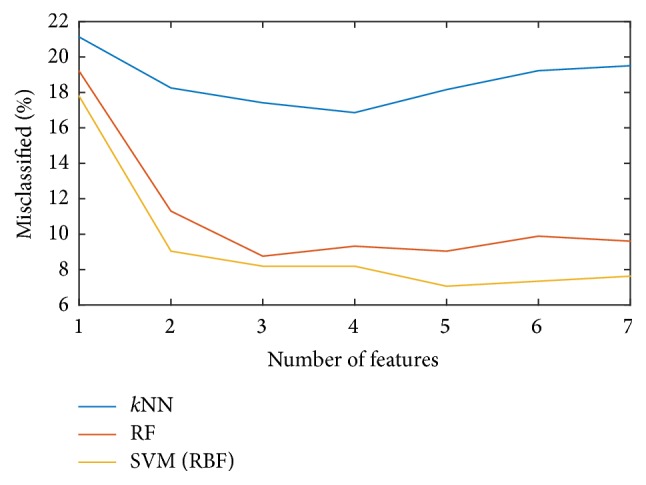
Error rates of classifiers using *k*NN, RF, and SVM method.

The classification accuracy of the classifier used in this study is also compared with that of two other common methods, *k* nearest neighbor (*k*NN) and Random Forest (RF). The *k*NN method calculates the Euclidean distance between the unknown sample's feature vector with other feature vectors in the training dataset and predicts the class of the unknown sample by analyzing a certain number (*k*) of the nearest neighbors [52]. RF is an ensemble-based learning algorithm which predicts the class of a new sample by averaging predictions of a set of tree-based classifiers [53]. Each classifier in RF is constructed by using a subset of randomly selected data points from the original dataset. For *k*NN, the number of neighbors used is set to 3. For RF, the size of the random subsets at a node is set to the square root of the number of features in the data. The accuracy for these 3 classifiers is shown in Figure 9. The SVM classifier with RBF kernel has the smallest classification error for our application.

It has been shown that considerably high classification accuracy can be achieved for our cell selection system by using SVM classifiers with Gaussian kernel. Further improvements in classification performances may be achieved by applying ensemble techniques to combine different individual classifiers [54]. The RF classifier is one example that combines a set of decision trees in order to improve the classification accuracy of a single decision tree. Different families of classifiers can also be combined to increase performance [55, 56]. In the future, different combination methods and diversification methods will be explored to generate new ensemble classifiers and the method will be evaluated in clinical trials.

## 5. Conclusions

In this study, we presented a method for classifying detected objects on raw buccal smear images into suitable and unsuitable objects. Using this method, cells that are folded, overlapped, damaged, or obscured by debris can be excluded from further analysis. The trained classifiers show good performance in distinguishing the two classes. This method provides a prescreening for automated cytological analysis based on nanocytology (PWS microscopy). The method was tested on buccal cytology but can easily be extended for evaluation of other types of cytological samples. This automated technique may prove to be a valuable method of cell selection, with particular relevance to translation in the clinic where clinical trials of PWS microscopy in lung cancer patients are due to begin shortly.

## Figures and Tables

**Table 1:** Description of features used for classifier development.

Type of feature	Number of features
Size and shape: describing the shape of the object	8
Histogram features: describing the grey scale distribution	11
Color: describing the staining color of the sample	9
Texture features 1: describing the general characteristics of grey scale texture	35
Texture features 2: describing textures based on Fourier transform and models	30
Topology: describing the surface morphology	7

**Table 2:** Top ranked features for the classifier.

Features	Definition	Note
Eccentricity	$\sqrt{1-\frac{b^{2}}{a^{2}}}$	*a*, *b*: semimajor and semiminor axes

Solidity	$\frac{A}{H}$	*A*: area of the shape region; *H*: convex hull area of the shape

Normalized kurtosis	$\frac{E{\left(x-\mu \right)}^{4}}{\sigma^{4}}$	*μ*: mean of *x*; *σ*: standard deviation of *x*

Normalized variance	$\frac{\sigma^{2}}{\mu^{2}}$	—

Entropy	−∑(*p∗*log⁡(*p*))	*p*: counts from intensity histogram

Energy	$E=\underset{i}{\sum }\underset{j}{\sum }{\left(M\left(i,j\right)\right)}^{2}$	*M* is the sum of the concurrence matrices with offset of 5 pixels in eight neighborhoods [36]

Variance of image filtered with Gabor filter [37]	$exp\left(-\frac{\left(\left(x^{2}/\sigma_{x}\right)+\left(y^{2}/\sigma_{y}\right)\right)}{2}\right)\cos\left(2\pi \mu \left(x\cos\theta +y\sin\theta \right)\right)$	*θ*: the orientation of the filter, averaged over 4 directions

**Table 3:** 

Features	Values	
	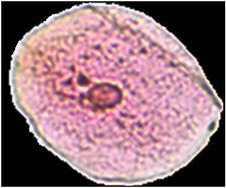	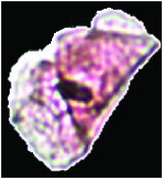

Eccentricity	0.6378	0.8905
Solidity	0.9532	0.9025
Normalized variance	0.01523	0.1294
Normalized kurtosis	4.495	2.026
Entropy	6.472	7.504
Energy	0.07223	0.007897
Variance of image filtered with Gabor filter	0.0002908	0.0003888

**Table 4:** Commonly used SVM kernel functions.

Kernel transform	Expression
Linear	〈*x*_*i*_, *x*_*j*_〉
Polynomial	(〈*x*_*i*_, *x*_*j*_〉 + *a*)^*d*^
Radial basis function	*e* ^−‖*x*_*i*_ − *x*_*j*_‖^2^/2*σ*^2^^
